# A greater focus on metals in biomedicine and neuroscience is needed

**DOI:** 10.1186/s40360-016-0095-4

**Published:** 2016-11-05

**Authors:** Anthony R. White

**Affiliations:** Department of Pathology, University of Melbourne, Parkville, VIC 3010 Australia

**Keywords:** Metals, Biometals, Neurodegeneration, Brain disorders, Neuroscience, Biomedical research

## Abstract

Metals have many essential functions in the brain and a large body of evidence supports important roles for altered metal stasis in many brain disorders. However, despite this evidence, acceptance of metals as key mediators of brain dysfunction is largely lacking in mainstream biomedicine. This editorial will outline the possible reasons for this and suggest potential means to improve the acceptance of metals as central players in brain disease.

## Main text

Diseases of the brain, whether they be psychiatric or neurodegenerative disorders, are a growing global health concern. As many of these disorders are associated with ageing we are seeing rapid increases in the number of patients, but this is not being matched by advances in understanding and treatment of these diseases. In fact, many potential drug candidates have recently shown little efficacy in advanced clinical trials for many neurodegenerative disorders. Clearly, we still have a lot to learn about the pathological processes of these complex brain disorders. Of particular concern is the absence of discussion in mainstream biomedicine and neuroscience regarding the central role metals have in brain function and disease. While bulk metals such as calcium and magnesium are well accepted as essential mediators of brain cell activity throughout the fields of medicine and neuroscience, other metals, particularly the transition elements, have not faired as well.

Metals such as copper, zinc, iron, selenium, manganese and others have critical roles in many brain cell processes including energy production, anti-oxidant activity, structural roles in proteins, modulation of nucleic acid processing, inflammation, neurotransmission and more. Maintaining the correct homeostasis of these metals is critical as any substantial change can lead to neurotoxic outcomes. The importance of metals to neuronal and glial function in health and their contribution to disease processes has been clearly demonstrated. However, mainstream biomedical research, which focuses heavily on genes, proteins and lipids, often overlooks the findings even when published in high impact journals. An example of this situation includes the failure of popular websites to list metals as key elements in neurodegenerative diseases alongside protein aggregation, oxidative stress, and inflammatory processes, yet metals have been demonstrated to have major roles in all of those pathogenic changes. Metals and their role in brain function and disease rarely receives coverage in keynote or plenary lectures or major symposia at leading international neuroscience or brain disease meetings, and is often absent from major reviews on neurodegeneration. Yet a search on *PubMed* for ‘metals and neurodegenerative diseases’ will quickly reveal that the number of publications is similar to neuroinflammation in neurodegenerative diseases, and there has been a large increase in publications on metals and the brain over the past decade.

There are likely several reasons for the continued absence of metals in mainstream discussion of brain function and disease. The issue may stem initially from limited focus on metals in undergraduate biomedicine programs, where the emphasis is on complex organic molecules. Metals tend to be covered more in chemistry and less in biology. Perhaps, the situation is not helped by the reference to many of these elements as ‘trace’ metals. While this term is certainly correct in terms of quantity, it belies the essential role many metals have in cellular and especially brain functions. Additionally, much of the research on metals has been performed within the framework of chemistry and biophysical methodologies, which may seem somewhat obscure to researchers of a biomedical background more used to dealing with PCR than EPR. Finally, an oft quoted view I have come across in reviews of manuscripts on metal changes in the brain is that because there are often changes to many metals simultaneously this represents a consequence and not a cause of other pathologies. This phenomenon however is rarely directed at multiple gene or protein changes and seems to stem from a lack of awareness of the complex inter-relationship between multiple metal homeostatic processes.

So how do we elevate metals to their rightful status as key players in brain function and disease? Much of this is up to us, we need to present our research in a context that clearly shows that metals are integral to all major brain activities and functions and play an important role in degenerative processes in brain disease. We need to advocate to educators that metals should receive the same attention as the protein transporters and chaperones that traffic metals within cells. Major advances in bio-analytical, imaging, and proteomic detection of metals and metal-complexed molecules will also help us to fit metals into key components of the brain’s machinery, whether they be protein, lipid or nucleic acid-associated pathways, rather than simply reporting on global metal changes.

Importantly, the publication of this special issue on *neurometals* in BMC Pharmacology and Toxicology is a timely recognition of the need to provide focused and highly visible platforms to disseminate the concept of metals in brain function and disease. This is an exciting opportunity to bring neurometals to the forefront of brain research and support our advocacy for metals as key players in all aspects of brain processes, from molecular activity to cognitive function. We hope that this special issue will provide a node for other researchers across diverse fields to access brain metal research and integrate this into their own programs. We will know we have succeeded when we regularly see metals as plenary topics in leading international neuroscience and brain disease meetings.

## Abbreviations

EPR: Electron paramagnetic resonance
PCR: Polymerase chain reaction

